# Age related increase in mTOR activity contributes to the pathological changes in ovarian surface epithelium

**DOI:** 10.18632/oncotarget.8468

**Published:** 2016-03-29

**Authors:** Preety Bajwa, Prathima B. Nagendra, Sarah Nielsen, Subhransu S. Sahoo, Amanda Bielanowicz, Janine M. Lombard, Erby J. Wilkinson, Richard A. Miller, Pradeep S. Tanwar

**Affiliations:** ^1^ Gynaecology Oncology Group, School of Biomedical Sciences and Pharmacy, New South Wales, Australia; ^2^ Hunter Cancer Biobank, New South Wales, Australia; ^3^ School of Medicine and Public Health, University of Newcastle, Callaghan, New South Wales, Australia; ^4^ Division of Gynaecology Oncology, Department of Medical Oncology, Calvary Mater Newcastle, Waratah, New South Wales, Australia; ^5^ Unit for Laboratory Animal Medicine, University of Michigan School of Medicine, Ann Arbor, MI, USA; ^6^ Department of Pathology and Geriatrics Center, University of Michigan, Ann Arbor, MI, USA

**Keywords:** ovarian aging, rapamycin, mTOR, ovary, OSE, Gerotarget

## Abstract

Ovarian cancer is a disease of older women. However, the molecular mechanisms of ovarian aging and their contribution to the pathogenesis of ovarian cancer are currently unclear. mTOR signalling is a major regulator of aging as suppression of this pathway extends lifespan in model organisms. Overactive mTOR signalling is present in up to 80% of ovarian cancer samples and is associated with poor prognosis. This study examined the role of mTOR signalling in age-associated changes in ovarian surface epithelium (OSE). Histological examination of ovaries from both aged mice and women revealed OSE cell hyperplasia, papillary growth and inclusion cysts. These pathological lesions expressed bonafide markers of ovarian cancer precursor lesions, Pax8 and Stathmin 1, and were presented with elevated mTOR signalling. To understand whether overactive mTOR signalling is responsible for the development of these pathological changes, we analysed ovaries of the *Pten* trangenic mice and found significant reduction in OSE lesions compared to controls. Furthermore, pharmacological suppression of mTOR signalling significantly decreased OSE hyperplasia in aged mice. Treatment with mTOR inhibitors reduced human ovarian cancer cell viability, proliferation and colony forming ability. Collectively, we have established the role of mTOR signalling in age-related OSE pathologies and initiation of ovarian cancer.

## INTRODUCTION

Age is a major risk factor for the development of epithelial ovarian cancer (OvCa). Every year approximately 238,700 new cases are diagnosed and 151,900 deaths are attributed to OvCa, worldwide [1]. The majority of OvCa patients are 50 years of age or older and postmenopausal [2]. Although the impact of aging in germ cell biology and fertility is well known [3], very little information is available regarding the contribution of ovarian aging to the pathogenesis of OvCa.

In mammalian ovary, germ cells are surrounded by the somatic cells to form a basic functional unit known as follicles. During every oestrous cycle, some of the early stage follicles, known as the primordial follicles, are recruited to grow and ultimately these developed follicles either undergo atresia or ovulation to release a mature egg for fertilization [4]. However, with age, mammalian ovaries progressively run out of follicles due to various reasons and females undergo menopause, a stage in which women do not regularly experience monthly oestrous cycles. In 1971, Fathalla proposed that repetitive rupture and repair of the ovarian surface epithelium (OSE) during the process of ovulation creates opportunities for accumulation of genetic aberrations leading to the abnormal growth of these cells [5]. This hypothesis explained high prevalence of OvCa in modern women and domestic egg-laying hens compared to other mammals [6]. Epidemiological association studies also provided support for Fathalla's hypothesis, whereby physiological conditions that suppress ovulation, such as pregnancy and breast-feeding, protected against OvCa [6-8]. Whereas, nulliparous women with a higher number of ovulatory cycles due to the absence of pregnancy and lactation, for example nuns, are more prone to developing OvCa compared to the general population [9].

In mouse models, increase in the frequency of ovulations either by hormonal treatments or by controlled exposure to the male mice leads to abnormal OSE cell proliferation and formation of inclusion cysts [10, 11]. However, these studies were conducted in relatively young mice with a limited follow up and therefore the pathogenic potential of these cysts is unknown. Interestingly, a higher number of inclusion cysts are observed in the contralateral ovaries of unilateral OvCa patients and in the ovaries of patients with hereditary predisposition to OvCa compared to general population [12, 13]. Assessment of genetic aberrations in ovaries of patients with OvCa, borderline tumours and non-neoplastic disease showed chromosomal alterations in OSE and inclusion cysts [14]. Moreover, a higher proportion of aneusomic cells were present in the inclusion cysts from OvCa and borderline tumour patients than controls [14], suggesting that these OSE-derived cysts have the potential to progress to OvCa. These findings are well supported by observations in genetically modified mouse models where, in addition to the Fallopian tube epithelium, genetic alterations in OSE causes development of OvCa that are phenotypically similar to human OvCa [15-17].

Incessant ovulation hypothesis and related work from many other laboratories have provided possible explanation for the development of ovarian epithelial inclusion cysts and their probable involvement in pathogenesis of OvCa but there are still many outstanding questions which need to be addressed for better understanding of this disease. Firstly, mice with germ cell deficiency also exhibit OSE hyperplasia and inclusion cysts, and develop ovarian epithelial tumours with advancing age [18]. Secondly, ovaries of women with anovulatory polycystic ovary syndrome also have inclusion cysts even though these women rarely or never ovulate [8]. Thirdly, inclusion cysts are more prevalent in aged human ovaries (> 60 years of age), even when there is no ovulation [19]. Collectively, these findings suggest that age associated changes in ovary/OSE contribute to the formation of inclusion cysts and consequently, OvCa.

The mammalian target of rapamycin (mTOR) pathway is involved in various cellular processes and inhibition of mTOR signalling has been shown to extend life span in several different species, including yeast, flies, worms, and mice, in part by suppressing age related pathologies and cancer [20-25]. Widespread genetic alterations in the mTOR pathway members are common in OvCa patients and comparable aberrations lead to the development of histopathologically similar tumours in mouse models [16]. On the basis of these studies, we hypothesised that age related increase in mTOR activity contributes to the development of OSE hyperplasia and inclusion cysts. In this study, we have shown hyperactivation of mTOR signalling in OSE lesions of the aged human and mouse ovaries, and inhibition of this signalling pathway suppressed development of these pathologies in mouse ovaries.

## RESULTS

### OSE hyperplasia in aged human and mouse ovaries

**Figure 1:**
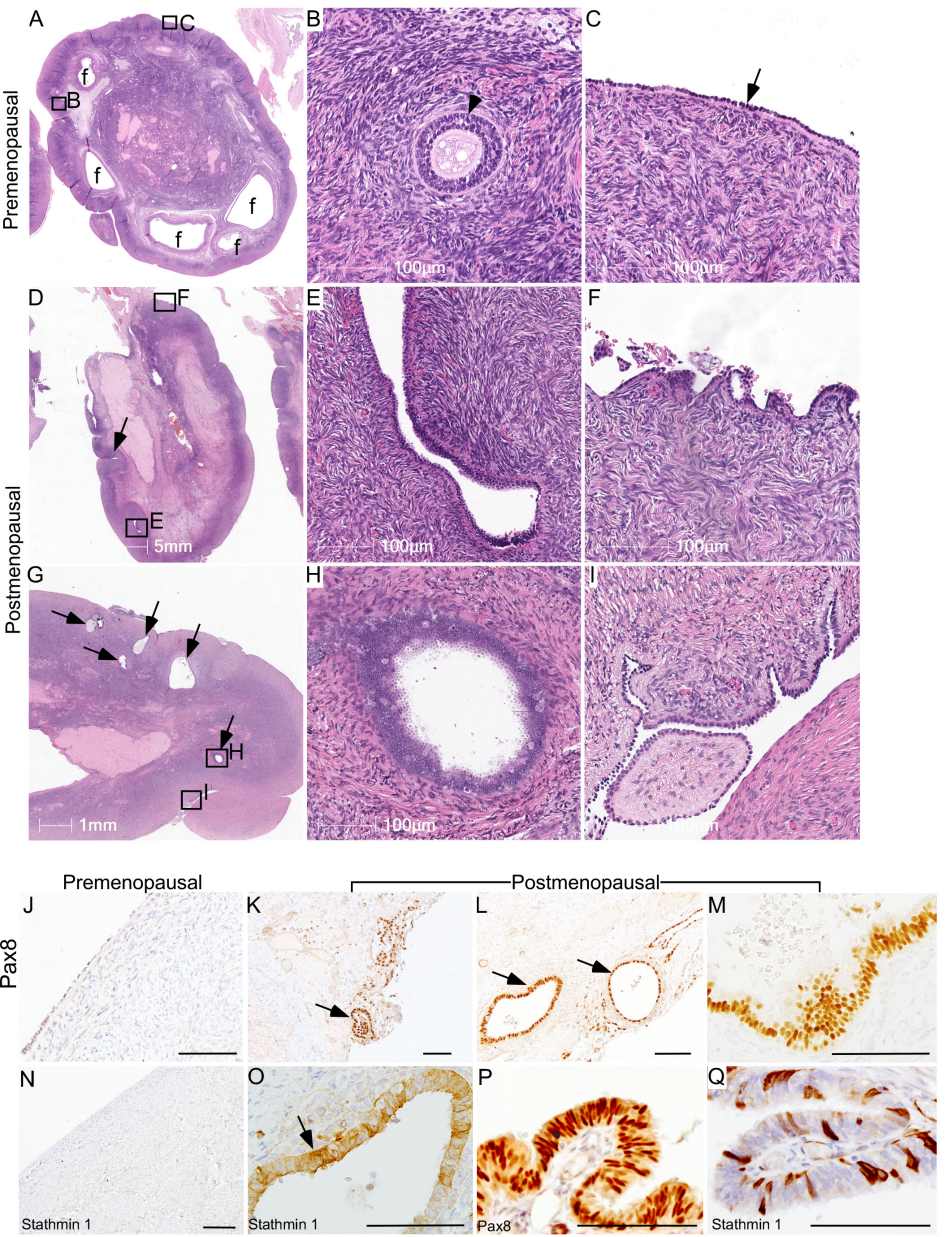
Ovarian surface epithelium hyperplasia and epithelial inclusion cysts in postmenopausal human ovaries **A.-C.** Representative histological sections of premenopausal human ovaries showing normal morphology with different sized follicles (marked with f) and a single layer of ovarian surface epithelium (arrow in panel C). A representative picture of a preantral follicle (arrowhead) is presented in Panel B. **B.** and **C.** are higher magnification images of boxed areas in panel A. **D.-I.** Examination of postmenopausal ovaries showing absence of follicles and displaying OSE cell hyperplasia with deep surface invaginations (arrow in panel D), abnormal papillary growth **F.**, inclusion cysts (E, arrows in panel G and a high magnification image in panel H), epithelial outgrowths and shedding **I.**. Boxed areas in **D.** and **G.** are presented at a higher magnification in **E.**, **F.**, **H.** and **I. K.-M.** PAX8 immunolocalization in abnormal epithelial lesions (arrows in panel **K.** and **L.**) of postmenopausal human ovaries. **O.** Inclusion cysts in a postmenopausal ovary were also positive for Stathmin 1 (arrow). OSE of control premenopausal ovaries stained negative for both PAX8 **J.** and Stathmin 1 **N.**. **P.** and **Q.** Fallopian tube epithelial cells were used as a positive control for both the markers. Bars: 100 μm, if not specified in a panel.

To understand how aging affects OSE cells, we examined human ovaries collected from pre- and post-menopausal women. Analysis of the premenopausal ovaries showed the presence of follicles in different stages of their development and a single layer of epithelial cells, known as OSE, covering the outer surface of these ovaries (Figure 1A-1C; *N* = 3). As expected, there were no follicles in the postmenopausal ovaries (Figure 1D-1I; *N* = 9). However, epithelial inclusion cysts (Figure 1E and 1H), OSE papillary and stratified growth (Figure 1F and 1I), and deep surface invaginations were clearly present in the postmenopausal ovaries (Figure 1D-1I), which were absent in the premenopausal ovaries (Figure 1A-1C). To evaluate if these epithelial lesions represent the precancerous state of ovarian cancer, we performed immunohistochemical localization of Pax8 and Stathmin 1, well-established markers of ovarian cancer precursor lesions and malignant disease [26, 27]. Expression of these two markers was absent in normal OSE cells of the premenopausal ovaries (Figure 1J and 1N). However, these markers were expressed by the epithelial inclusion cysts and abnormal OSE growths present in the postmenopausal ovaries (Figure 1K, 1L, 1M and 1O), suggesting that OSE in aged ovaries undergo metaplastic changes to acquire some of the features of ovarian cancer precursor lesions. Fallopian tube sections were used as positive controls as both the markers are known to be expressed in this tissue (Figure 1P and 1Q). Postmenopausal ovarian tissue slides that were exposed to IgG showed no staining and were used as negative controls (Supp. Figure 1).

**Figure 2:**
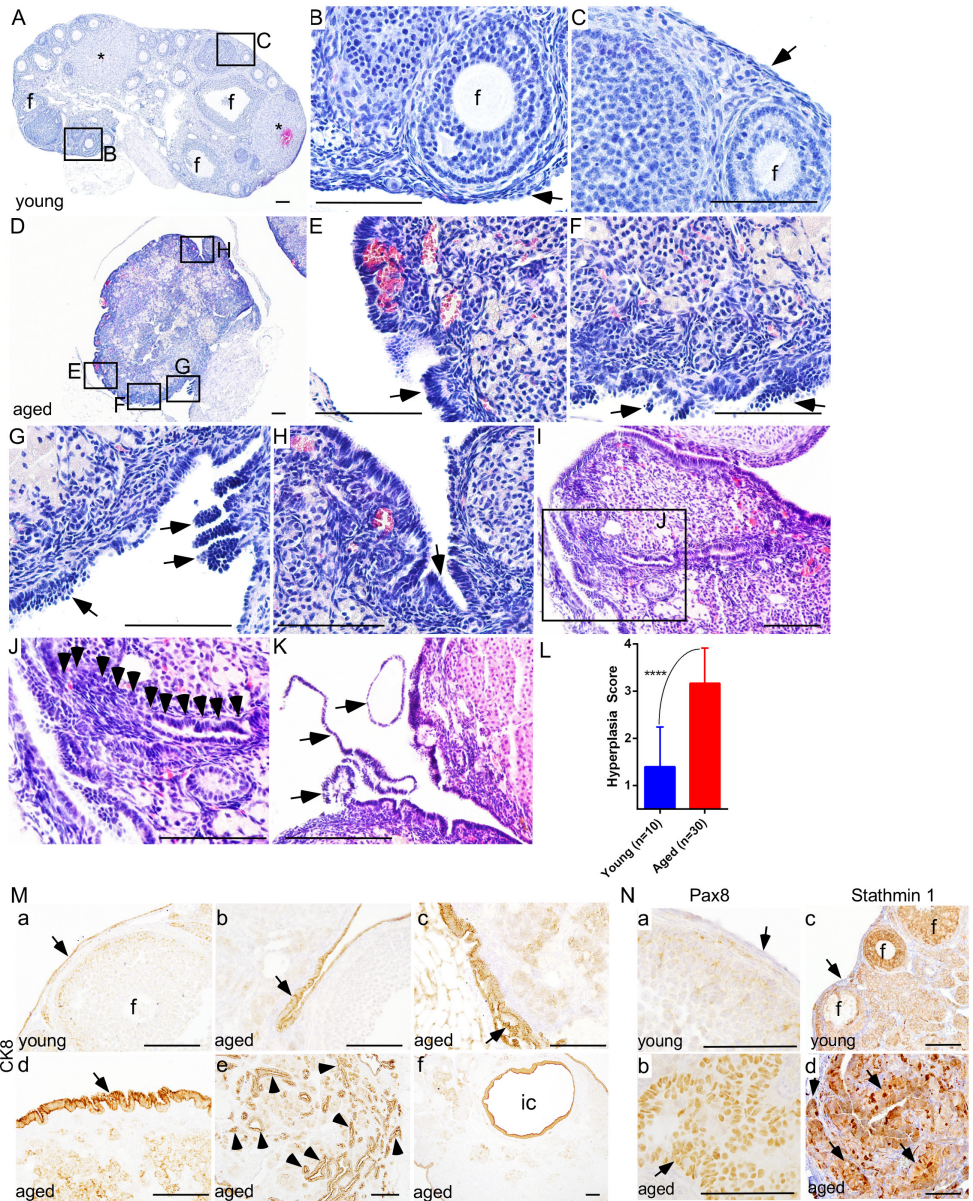
Deregulated growth of ovarian surface epithelium in aged mouse ovaries **A.-C.** Histological analysis of a young mouse ovary (8 wk) showing follicles (B; marked with f), corpora lutea (asterisks in panel A), and a single flattened layer of ovarian surface epithelial cells (C; arrow). **D.** An abnormal looking hyperplastic ovary of aged mice. Higher magnification images of boxed areas in panel D revealed irregular morphology and ovarian surface epithelial cell hyperplasia (arrows in **E.** and **F.**), epithelial multilayering and abnormal papillary growth (arrows in **G.**), and epithelial invaginations into stroma **H. I.** and **J.** OSE cells in an aged ovary infiltrating (arrowheads in panel J) into the stroma forming inclusion cysts and glandular structures. Panel J is a higher magnification picture of boxed area in panel I. Extensive epithelial shedding and outgrowths (arrows in panel **K.**) in aged ovaries. **L.** Significant increase in ovarian surface epithelial hyperplasia score of ovaries of the aged mice compared to young controls. **M.** Epithelial origin of abnormal lesions confirmed by CK8 staining. (**M.**, a) Normal CK8-postive OSE cells (arrow) were seen in young mouse ovary. (**M.**, b-f) CK8 expression was observed in abnormal invasive and hyperplastic growths (arrow in panel b-d, arrowheads in panel e) of OSE in aged mice. (**M.**, f) CK8-positive inclusion cyst (ic) present in an aged ovary. (**N.**, b and d) PAX8 and Stathmin 1 expression in invasive and hyperplastic epithelial growths (arrow) of aged mice ovaries. (**N.**, a and c) Ovarian surface epithelium (arrow) of young mouse ovary was negative for PAX8 and Stathmin 1 staining. (**N.**, c) Stathmin 1 was expressed by the somatic cells of follicles (marked with f) in young ovaries. Bars: 100 μm.

To confirm whether similar changes occur in mouse ovaries, we aged 50 C57BL/6 mice for 22 months and tissues were collected at regular intervals. Histological examination of young mouse ovaries (Age: 8 weeks; *N* = 10) showed follicles, corpora lutea, and a single layer of flattened-to-cuboidal OSE cells (Figure 2A-2C). Similar to the post-menopausal human ovaries, aged mouse ovaries exhibited features such as OSE hyperplasia (Figure 2E and 2F), papillary growth (Figure 2G), deep surface invaginations (Figure 2H), inclusion cysts (Figure 2I and 2J) and shedding (Figure 2K). Staining with cytokeratin 8 (CK8), a well-known marker of epithelial cells [16], validated the epithelial origin of these pathological lesions (Figure 2M).

Ovarian inclusion cysts have been proposed to derive from the deep invaginations of OSE cells or entrapment of the fallopian tube epithelial cells during the process of repetitive ovulatory wound and repair [19]. To ascertain the possible cell of origin of epithelial inclusion cysts, we performed extensive serial sectioning of the aged mouse ovaries and found that these cysts are connected to the OSE cells (Figure 2I and 2J), suggesting that epithelial inclusion cysts in the aged ovaries are coming from the invaginations of the hyperplastic OSE cells. Using a well-established method of scoring OSE hyperplasia (Supp. Figure 2), as described in [10], we demonstrated that aged mouse ovaries (Age: 16 months; *N* = 30) have a significantly higher hyperplasia score compared to young ovaries (Figure 2L). To evaluate if abnormal OSE growths in aged mouse ovaries are similar to the epithelial lesions observed in postmenopausal human ovaries, we analysed expression of Pax8 and Stathmin 1 and found expression of both of these markers in abnormal epithelial growths of aged mouse ovaries (Figure 2N). In summary, these results suggest that OSE hyperplasia and inclusion cysts development occurs during ovarian aging, and these abnormal growths express markers of ovarian cancer precursor lesions.

### Hyperactive mTOR signalling in OSE of aged human and mouse ovaries

**Figure 3:**
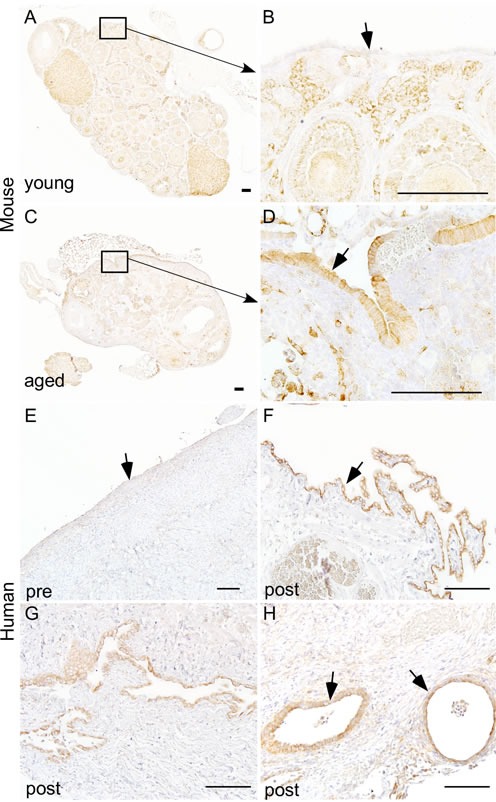
Hyperactive mTOR signalling in ovarian surface epithelium of aged human and mouse ovaries Increased expression of pS6, a marker for mTOR activation, was observed in hyperplastic ovarian surface epithelium (arrow in panel **D.**) of aged mouse ovaries **C.-D.** and papillary growths of ovarian surface epithelium of postmenopausal human ovaries (arrow in panel **F.**). Increased expression of pS6 was also observed in invasive epithelial growths **G.** and inclusion cysts (arrows in panel H) of postmenopausal human ovaries. Ovarian surface epithelium of young mouse **A.-B.** and human ovary **E.** showed negative staining for pS6. Panel **B.** and **D.** represent higher magnification images of boxed areas in panel **A.** and **C.** Bars: 100 μm.

We [16] and others [28, 29] have shown that overactivation of mTOR signalling occurs in approximately 80% of human ovarian carcinomas. In mouse ovary, constitutive activation of PI3K/mTOR signalling results in the development of similar tumours confirming the role of this pathway in pathogenesis of ovarian cancer. As OvCa is a disease of aged women [6], we hypothesised that mTOR activation contributes to the development of pathological changes in the OSE of aged ovaries. To test this hypothesis, we analysed the expression of phosphorylated form of S6 ribosomal protein (pS6), a known marker of active mTOR signalling [16], in both young and aged ovaries (Figure 3). In young mouse ovaries, pS6 is highly expressed in the somatic cells of the ovary but absent in OSE (Figure 3A and 3B). However, both OSE and somatic cells of the aged mouse ovaries showed expression of pS6 protein (Figure 3C and 3D). A similar expression pattern was observed in human ovaries where pS6 was localised in OSE and somatic cells of the postmenopausal ovaries but only present in the somatic cells of the premenopausal ovaries (Figure 3E-3H and Supp. Figure 3). In conclusion, our expression analysis revealed that overactivation of mTOR signalling occurs in OSE during ovarian aging.

### Genetic suppression of mTOR signalling inhibits OSE hyperplasia in aged ovaries

**Figure 4:**
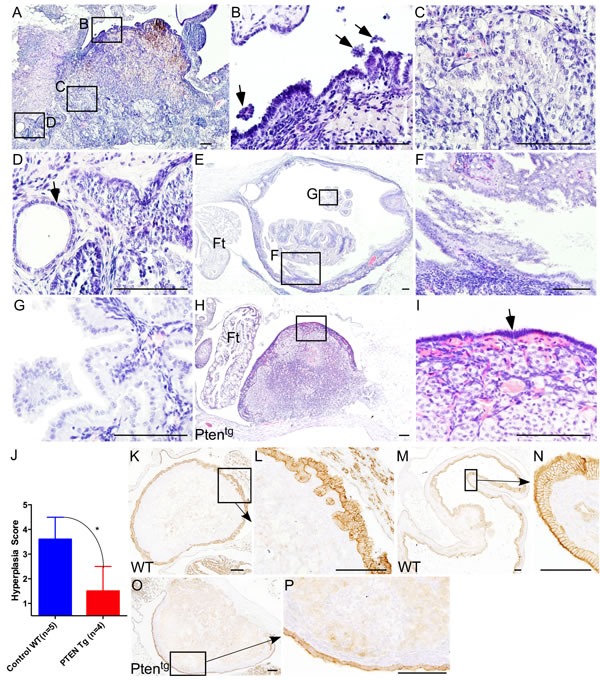
Overexpression of Pten inhibits ovarian surface epithelial hyperplasia in aged ovaries **A.-G.** Haematoxylin and eosin-stained sections of aged control mice ovaries. Boxed areas in panel A are shown at a higher magnification in panel **B.-D.** Ovarian surface epithelial cell hyperplasia and shedding (B; arrows), invasive epithelial growths **C.**, and inclusion cysts (D; arrow) in age matched control ovaries. **E.-G.** Papillary growths inside aged ovaries that are distinct from the fallopian tube epithelium. Panel **F.** and **G.** are high magnification images of boxed areas in panel E. **H.** Ovaries of the Pten^tg^ mice showed relatively normal morphology and are covered by single layer of ovarian surface epithelial cells, presented at a higher magnification and marked with an arrow in panel **I. J.** Significant decrease in hyperplasia score of the Pten^tg^ mice ovaries compared to age-matched controls. **K.-N.** Ovarian surface epithelium hyperplasia and invasive epithelial growths were confirmed by CK8 staining in wild type aged control mouse ovaries. CK8-positive papillary growths and deep surface invaginations are shown in panel L and N, respectively. **O.-P.** Normal looking CK8-positive OSE cells in Pten^tg^ mice ovaries. Ft: Fallopian tube. Bars: 100 μm.

A previous study has shown that overexpression of *Pten*, a negative regulator of PI3K/mTOR signalling, significantly extends lifespan of mice, partially by decreasing incidences of cancer in aged mice [30]. For this study, we collected ovaries from aged *Pten* transgenic (Pten^tg^) and wild type (WT) control mice (*N* = 5/each; age: 26-27 months). Examination of WT mouse ovaries revealed OSE shedding, inclusion cysts and invasive papillary growths (Figure 4A-4G). Next, we serially sectioned the ovaries and the Fallopian tubes of the WT mice and showed that the papillary growths within the WT ovaries are connected with OSE and are distinct from the fallopian tube epithelial cells (Figure 4E-4G), suggesting that these abnormal epithelial growths are extensions of invasive OSE cells. Analysis of aged Pten^tg^ ovaries depicted a single layer of OSE cells around the whole ovarian surface with no evidence of invasive growth (Figure 4H and 4I), which was similar to young mouse ovaries (Figure 2A-2C). The ovaries of the Pten^tg^ mice had a significantly lower OSE hyperplasia score compared to the WT controls (Figure 4J). Immunolocalization of CK8, Pax8 and Stathmin 1 confirmed phenotypic changes in the OSE of Pten^tg^ and control ovaries (Figure 4K-4P and Supp. Figure 4). As expected, compared to controls, lower expression of pS6 protein was observed in the Pten^tg^ ovaries (Supp. Figure 4). The mammalian ovaries progressively lose their follicles through ovulation or apoptosis and eventually run out of these germ cell units leading to menopause [4]. The majority of the follicles are lost by one year of age in mice and by 50 years of age in humans [3]. Consistently, no follicles were observed in the ovaries of aged** Pten^tg^ and control mice (Figure 4A and 4H). These results establish that suppression of mTOR signalling by overexpression of *Pten* is sufficient to inhibit pathological changes in aged OSE cells.

### Pharmacological inhibition of mTOR signalling suppresses age-associated changes in OSE

**Figure 5:**
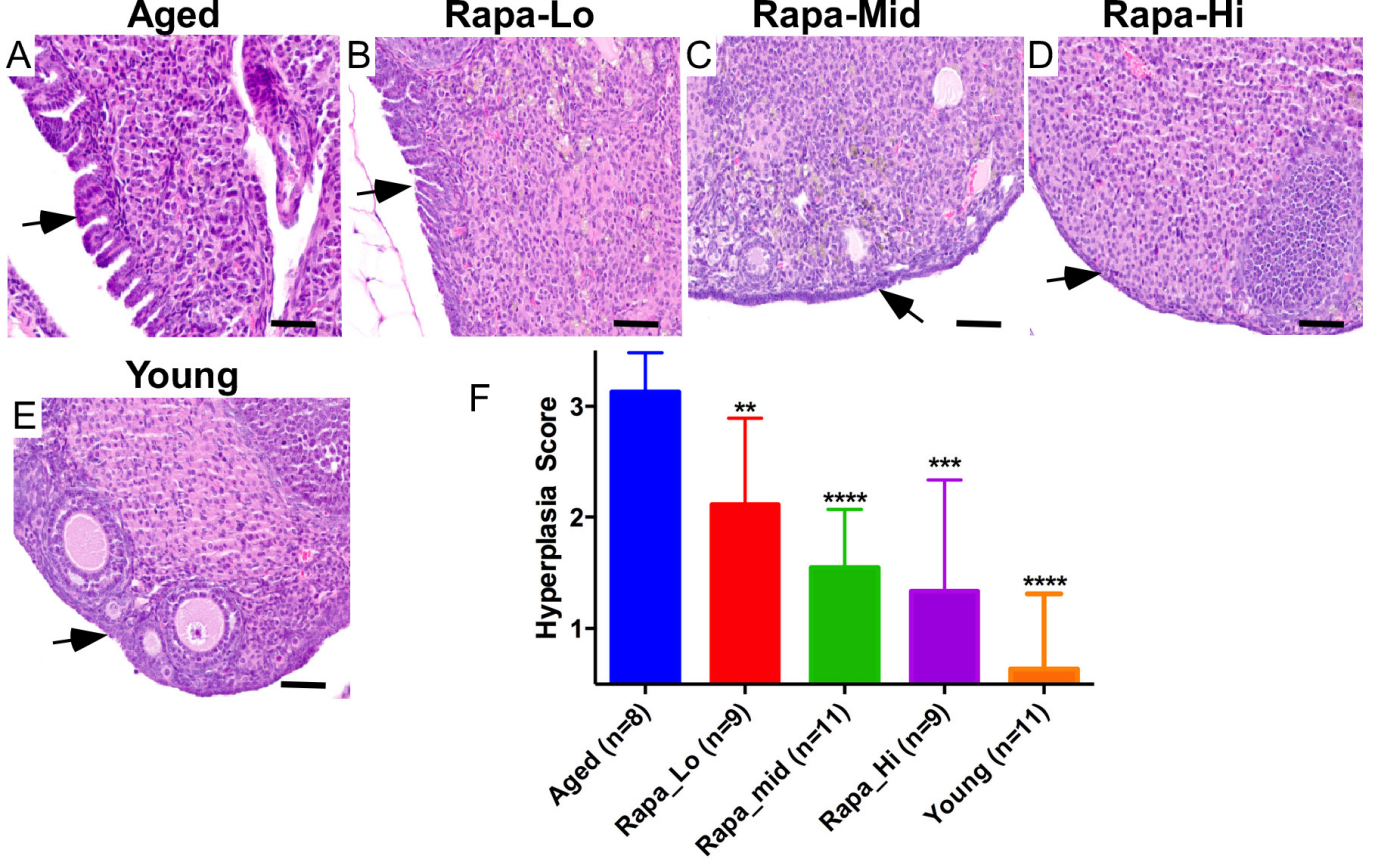
Chronic rapamycin treatment suppresses age-associated pathological changes in ovarian surface epithelium **A.** Aged control mice showing abnormal papillary epithelial growth (arrow). **B.-D.** Treatment with rapamycin decreased hyperplastic growth (arrows) of ovarian surface epithelium in a dose-dependent manner. Histology of ovaries of the aged mice treated with low dose **B.**, medium dose **C.** and high dose **D.** of rapamycin. In panel **E.** is a representative section of the ovary from young control mice with normal ovarian surface epithelial cell lining. **F.** Hyperplasia score confirmed significant decrease in abnormal epithelial growth of the aged ovaries treated with increasing doses of rapamycin. Bars: 100 μm.

Several independent studies have established that treatment with rapamycin, an inhibitor of mTOR kinase, extends lifespan by delaying the onset of age related pathological disorders including cancer [21-23, 25, 31, 32]. To test if the inhibition of mTOR signalling using an mTOR inhibitor will suppress the pathological changes in aged ovaries, we collected ovaries from genetically heterogeneous mice that had been treated with rapamycin from 9 months of age at three different doses (4.7, 14, or 42 parts per million in food) for 13 months. Consistent with previous results (Figure 2), histological examination of ovaries from 22-month-old untreated mice revealed OSE hyperplasia and papillary growth (Figure 5A; *N* = 8). Rapamycin treatment significantly suppressed these pathological changes in OSE cells in a dose dependent manner (Figure 5B-5D and 5F). The highest dose of rapamycin (42ppm) was most effective in inhibiting epithelial hyperplasia and OSE of the ovaries belonging to this treatment group were similar to young ovaries (Figure 5D and 5E). Overall, these results showed that chronic suppression of mTOR signalling inhibits age related incidences of OSE hyperplasia, and therapeutic targeting of this pathway might be an effective strategy for the prevention and/or treatment of OvCa.

### mTOR inhibitors suppress the growth of human OvCa cells

**Figure 6:**
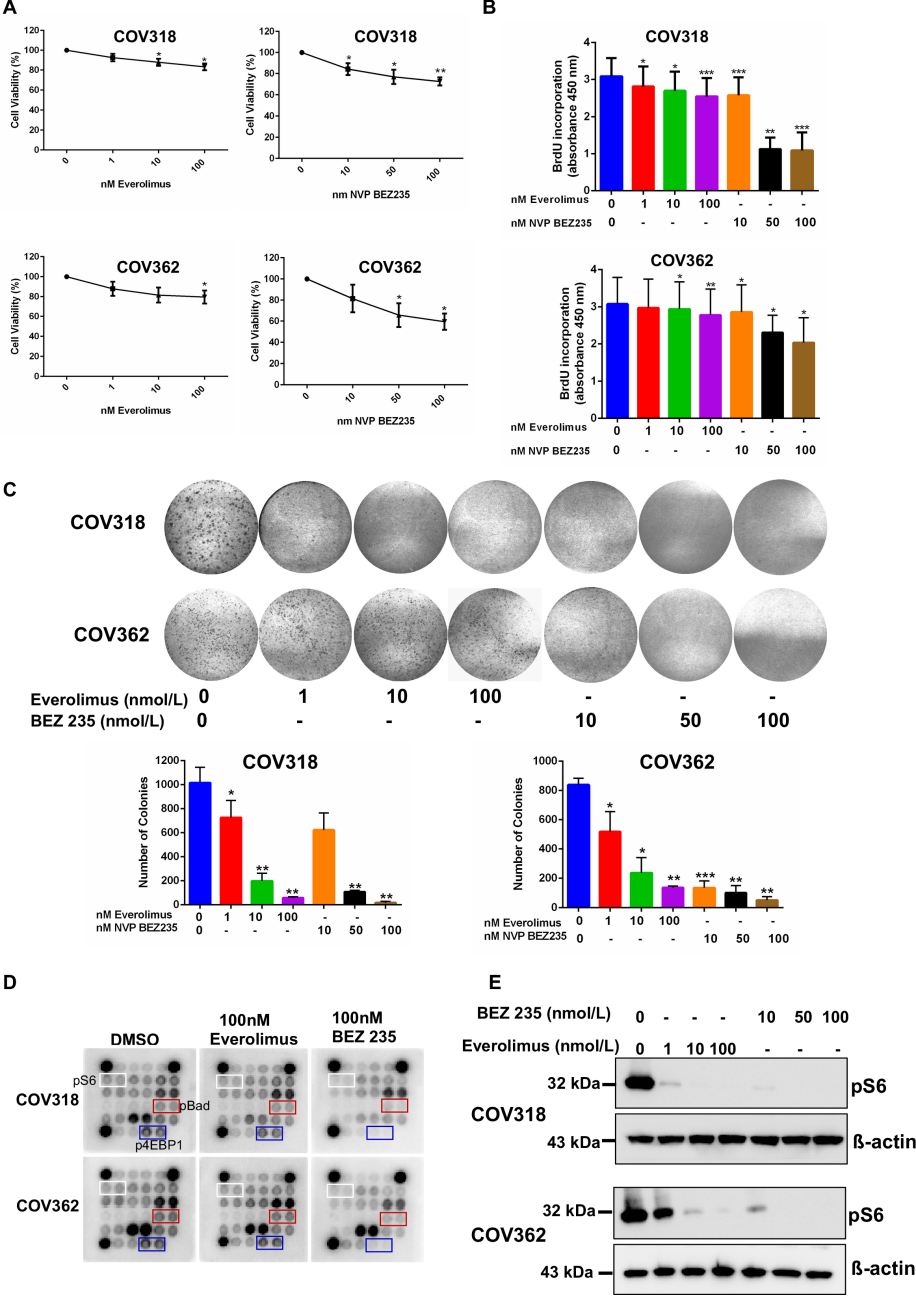
mTOR inhibitors suppress the growth of ovarian cancer cells **A.** COV318 and COV362 cells were treated with increasing doses of mTOR inhibitors, Everolimus and NVP BEZ235. Forty-eight hours later, cell viability was assessed using cell viability assay kit from Promega. The data shown are mean ±SEM of three individual experiments. *P < 0.05, **P < 0.01, Student's *t*-test. **B.** Cells treated with mTOR inhibitors were subjected to BrdU incorporation assay. The data represents mean ±SEM of three individual experiments. *P < 0.05, **P < 0.01, ***P < 0.001, Student's *t*-test. **C.** COV318 and COV362 cells treated with increasing dose of mTOR inhibitors were subjected to clonogenic assays. The data shown are representative of three individual experiments. Pictures were taken at the same magnification. *P < 0.05, **P < 0.01, ***P < 0.001, Student's *t*-test. **D.** COV318 and COV362 cells were treated with DMSO control or 100 nmol/L of Everolimus or BEZ235. Forty-eight hours later, whole cell lysates were subjected to AKT/mTOR signalling antibody protein array. **E.** Whole cell lysates from COV318 and COV362 cells treated with increasing doses of Everolimus and BEZ235 were subjected to western blot analysis for pS6. The data shown are representative of three individual western blot analyses. β-actin was used as a loading control.

OvCa is a disease that mainly occurs in postmenopausal women [6]. Our analysis of aged human and mouse ovaries found epithelial lesions with hyperactive mTOR signalling that share histopathological features of the OvCa precursor lesions and malignant disease (Figures 1, 2, 3). To test whether pharmacological suppression of mTOR signalling using two FDA-approved drugs (Everolimus and BEZ235) will suppress the growth of OvCa, we treated OvCa cell lines (COV318 and COV362) with the different doses of these two mTOR inhibitors (Figure 6). COV318 and COV362 cells were selected based upon their genetic similarity to human OvCa [33]. Treatment with Everolimus or BEZ235 decreased cell viability of these OvCa cell lines in a dose dependent manner (Figure 6A). Assessment of cell proliferation using 5-bromo-2′-deoxyuridine (BrdU) incorporation and colony forming assays revealed significant decrease in OvCa cell proliferation upon exposure with mTOR inhibitors (Figure 6B and 6C). To confirm the efficacy of these inhibitors in suppressing mTOR activity, we performed AKT/mTOR protein array and showed that the treatment with Everolimus or NVP BEZ235 (100nmol/L) leads to the reduction in expression of the active form of the key downstream target proteins (pS6, p4E binding protein 1 and pBad) of this pathway (Figure 6D). To validate our AKT/mTOR array data, we performed western blot analysis for pS6 on protein extracts collected from COV318 and COV362 cells treated with increasing doses of Everolimus or BEZ235. Both inhibitors decreased the levels of pS6 protein (Figure 6E). Collectively, these findings showed that suppression of mTOR signalling decreases viability and proliferation of OvCa cells.

## DISCUSSION

PI3K/mTOR signalling is a key regulator of the major cellular processes such as cell proliferation and cell growth, and deregulation of this signalling pathway contributes to the pathogenesis of various diseases such as cancer, diabetes, obesity, hereditary diseases, and neurodegenerative disorders [34]. Studies in several different model systems have provided evidence that mTOR signalling is one of the main regulators of ageing process and genetic or pharmacological modulation of this signalling pathway affects lifespan [20, 32, 35]. Genetic deletion of the *mTor* or overexpression of the *tuberous sclerosis 1/2* (Tsc1/2) gene significantly increases mean life span in both worms and flies [20]. Similarly, pharmacological suppression of mTOR signalling using rapamycin or loss of the *mTor* or *S6K1* gene extends mouse lifespan [22, 25, 31, 36, 37]. These findings coupled with observations that the constitutive activation of mTOR signalling decreases [38] mean lifespan established that counteracting age related increase in mTOR signalling is one of the keys to controlling ageing. However, some recent studies have shown an opposite trend, where mTOR activity is decreased when determined in whole tissue samples of the aged mice compared young controls [39, 40]. For example, western blot analysis of pS6 protein in liver, muscle and fat tissue samples collected from young and aged C57BL/6J mice showed decrease in mTOR activity with aging [39]. Our examination of mTOR activity by measuring pS6 protein expression showed higher level of mTOR activity in young ovaries compared to aged ovaries, consistent with the idea that mTOR signalling plays an important role in germ and follicular development and young ovaries are filled with these structures, which are nearly absent in aged ovaries (Figures 2 and 3). However, OSE of the aged human and mouse ovaries showed strong expression of pS6 protein that was almost absent in the OSE of young ovaries (Figure 3). Our data is supported by the observations in fasting aged mice where the level of hepatic pS6 protein increases with age [41]. These findings suggest that changes in the level of mTOR activity with aging are distinct in each cell type and that assessment of mTOR signalling in a particular cell type might provide much more useful information compared to the whole tissue-based approaches.

OvCa is a very heterogeneous disease. There are three major types of OvCa: epithelial carcinomas, germ cell tumours, and stromal tumours [8]. The epithelial carcinomas represent the most malignant and common form of OvCa. Approximately 80% of the epithelial OvCa patients presented with overactivation of the mTOR pathway [16]. Constitutive activation of mTOR signalling by the deletion of the *Lkb1*/*Tsc1*/*Tsc2* gene in both OSE and stromal cells causes OSE hyperplasia and epithelial OvCa but no stromal tumours [16]. Similarly, overactivation of this signalling pathway in germ cells leads to premature germ cell loss and ovarian insufficiency [42]. However, no germ cell tumours were observed in these mice [42]. These findings imply that each cell type in an organ shows differential response to hyperactive mTOR signalling and high mTOR activity in a particular cell type rather than the whole organ is responsible for age related pathologies.

During each ovarian cycle, few primordial follicles are activated to grow, and these follicles under the influence of gonadotropins go on to develop to a preovulatory stage where they are ready to release mature eggs for fertilization. The process of primordial follicle recruitment is quite important for female fertility as any aberrations in this process lead to infertility [42]. Intra-follicular mTOR signalling is a key regulator of primordial follicle recruitment and growth. Sustained activation of mTOR in germ cells by genetic ablation of the *Pten*/*Tsc1*/*Tsc2* causes untimely recruitment of primordial follicles leading to premature ovarian failure and infertility [43]. This raises a possibility that the suppression of mTOR signalling might inhibit primordial follicle activation and consequently, delay menopause. However, examination of ovaries from the Pten^tg^ and rapamycin treated mice revealed no differences in follicle/stromal cells compared to control groups (Figures 4 and 5). This suggests that there are other compensatory mechanisms involved in the activation of primordial follicles.

Crosstalk between gonads and the hypothalamus-pituitary axis is an important determinant of mammalian fertility. Both hypothalamus and pituitary gland secrete hormones that affect ovarian function. In turn, ovarian hormones operate in feedback loop to regulate secretions from the hypothalamus-pituitary axis [4]. During aging, ovaries are almost devoid of follicles and endocrine feedback loop of the ovary is no longer operational leading to excessive secretion of gonadotropins, follicle-stimulating hormone (FSH) and luteinising hormone (LH). As gonadotropin receptors are present on OSE cells, these hormones are proposed to stimulate the growth of OSE and aid in development of inclusion cysts [8, 44]. In cell culture, both FSH and LH stimulate the growth of OSE cells by up regulating PI3K/mTOR signalling, and suppression of this signalling pathway inhibits gonadotropin-induced proliferation of OSE cells [44, 45]. This indicates that gonadotropins act through PI3K/mTOR signalling in regulating OSE growth, and similar mechanisms might be operational *in vivo* in aged ovaries.

Aging is a major risk factor for many solid cancers [46]. Lifestyle changes and pharmaceutical-based approaches that extend lifespan by counteracting the age-related pathological changes are also known to retard tumor development [21-23, 46-48]. We have previously shown that treatment with rapamycin extends mouse lifespan even when started as late as 20 months of age [31], suggesting that it may provide benefits related to mTOR action in growth or transformation of many different kinds of tumors, not just ovarian tumors. In summary, our examination of aged human and mouse ovaries showed hyperactivation of mTOR signalling in OSE pathological lesions. Suppression of mTOR signalling inhibited the development of these lesions in the Pten^tg^ and rapamycin treated mice. Treatment of human OvCa cells with mTOR inhibitors significantly decreased the growth and viability of these cells suggesting that targeting the mTOR pathway might be an effective therapeutic strategy for preventing OvCa.

## MATERIALS AND METHODS

### Mouse genetics and husbandry

Mice used in the present study were housed under standard animal housing conditions. All procedures for mice experimentation were approved by the Animal Care and Ethics Committee at the University of Newcastle. Generation and characterization of *Pten^tg^* mice is described in [30]. Rapamycin treatment in genetically heterogeneous mice is described by us in detail [32]. 50 C57BL/6 were aged for 22 months and tissues were collected at regular intervals.

### Human ovarian tissue samples

Human ovarian tissue samples from 12 patients were obtained from the Hunter Cancer Tissue Biobank using a protocol approved by the Institutional Human Research Ethics Committee at the University of Newcastle.

### Cell lines, reagents and culture conditions

The human ovarian cancer cells COV318 and COV362 (Sigma, MO, USA) were grown at 37°C in Dulbecco's Modified Eagle Medium supplemented with 10% fetal bovine serum (FBS), L-glutamine, and pencillin/streptomycin in a humidified atmosphere containing 5% CO_2_. Everolimus (RAD001) and NVP-BEZ235 were obtained from Selleckchem (Provided by Sapphire Biosciences, NSW, Australia). Short tandem repeat profiling and mycoplasma testing (MycoAlert^TM^ Plus Mycoplasma detection kit, Lonza, MD, USA) were conducted at regular intervals for the quality control of cell culture conditions and validation of these cell lines.

### Histology and Immunohistochemistry (IHC)

For histological analyses, ovaries were fixed in 10% formalin solution (Sigma, MO, USA) overnight at 4°C and then transferred to 70% ethanol until processing. The fixed tissues were dehydrated in a graded ethanol series, cleared in xylene, and embedded in paraffin wax. Embedded tissue samples were sectioned at 6 μm and mounted on slides. Haematoxylin and eosin (H&E) staining and IHC were performed using standard protocols [16]. Briefly, for IHC, antigen retrieval was performed in 1mM EDTA buffer (0.05% Tween-20, pH 8) followed by the endogenous peroxidase block using 3% (v/v) hydrogen peroxide in absolute methanol. Tissue sections were then blocked in blocking solution (5% Goat Serum in TBS, 0.1% Triton X-100) for 1 hr at room temperature. Following this, tissue sections were incubated overnight at 4°C with normal IgG or following primary antibodies: Stathmin 1 (1:2000), Phospho-S6 Ribosomal protein Ser235/236 (1:400; Cell Signalling Technologies, MA, USA), Pax8 (1:500, Proteintech, IL, USA) and CK8 (Developmental Studies Hybridoma Bank, IA, USA). Biotinylated secondary antibodies (Jackson ImmunoResearch Labs, PA, USA or Thermo Fischer Scientific, Australia) were used followed by incubation with horseradish peroxidase-conjugated streptavidin (Thermo Fischer Scientific). Sections were then exposed to Diaminobenzidine (DAB, Sigma) to develop colour. Sections were counterstained with hematoxylin. Images were photographed using Olympus DP72 microscope and the Aperio Scanscope slide scanner. The gain and exposure time were set constant across tissue samples.

### Hyperplasia scoring of mouse ovaries

For scoring OSE hyperplasia, histological sections of a mouse ovary were divided into four anatomical locations: hilus, perpendicular to the hilus, opposite from the hilus and centre of the ovary (as shown in Supp. Figure 2). Based upon the presence of morphological lesions or hyperplasia, a +1 score was assigned to each location. An overall score > 2 was considered as positive for hyperplasia.

### Cell proliferation assay

Cell proliferation assays were performed using BrDU cell proliferation assay kit (Cell Signaling Technology) as per the manufacturer's instructions. Briefly, 2500 cells/well were seeded onto 96-well plates and allowed to grow for 24 h followed by drug treatments. BrdU (10μmol/L) was added and cells were incubated for another 24 h before assay was carried out. Absorbance was read at 450 nm using SpectraMax microplate reader (Molecular Devices, CA, USA).

### Cell viability

Cell viability was quantitated using a Cell viability assay kit (Promega, NSW, Australia). Briefly, Cells were seeded at 5,000 cells per well onto 96-well culture plates and allowed to grow for 24 h followed by desired drug treatments. Post 48 hour treatment, cells were incubated with CellTiter-Blue^®^ reagent for 1 hour at 37°C. The fluorescent signal was recorded using the FLUOstar OPTIMA (BMG Labtech, VIC, Australia).

### Clonogenic assays

Cells were seeded at 2500 cells/ well onto 6-well culture plates and allowed to grow for 24 h followed by drug treatments. Cells were then allowed to grow for another 8 days before fixation with 70% ethanol and staining with 0.5% crystal violet. The images were captured with a Bio-rad VersaDoc™ image system (Bio-Rad, Gladsville, NSW). Colonies were counted using the National Institute of Health image J software.

### Western blot analyses and Akt signalling antibody array

Protein extracts from COV318 and COV362 treated with different concentrations of Everolimus or BEZ235 were prepared in ice-cold radioimmunoprecipitation assay buffer (RIPA) supplemented with protease and phosphatase inhibitors. Equal amounts of protein were loaded and separated by 10% SDS-PAGE gel and thereafter transferred to nitrocellulose membrane. Afterwards, the membrane was blocked in 5% milk (w/v) in Tris-buffered saline/Tween 20 for 1 hr at room temperature and then incubated overnight at 4°C with rabbit mAb Phospho-S6 Ribosomal protein (1:2000 in 2.5% w/v BSA, 1xTBS, 0.1% Tween-20; Cell Signalling Technologies). This was followed by incubation with secondary horseradish peroxidase-conjugated anti-rabbit antibody (Jackson ImmunoResearch, West Grove, PA) for 1hr at RT. βactin was used as a loading control. Akt/mTOR signalling antibody array was performed as per manufacturer's instructions (Cell Signalling Technologies).

### Statistical analysis

Statistical analyses were performed using GraphPad Prism 6.0 (Graphpad Software, San Diego, CA). Values are expressed as mean± SEM. The Student *t* test was used to calculate differences between the groups (N≥3/group), and *p* values ≤ 0.05 were considered statistically significant.

## SUPPLEMENTARY MATERIAL FIGURES


