# Supplementary material for: Age related increase in mTOR activity contributes to the pathological changes in ovarian surface epithelium
Source: Oncotarget. 2016 Mar 29;7(15):19214–27. doi: 10.18632/oncotarget.8468 (PMC4991377; doi:10.18632/oncotarget.8468)
Supplement: Supplementary file 1 [file oncotarget-07-19214-s001.pdf]

Age related increase in mTOR activity contributes to the pathological changes in ovarian surface epithelium

Supplementary Material

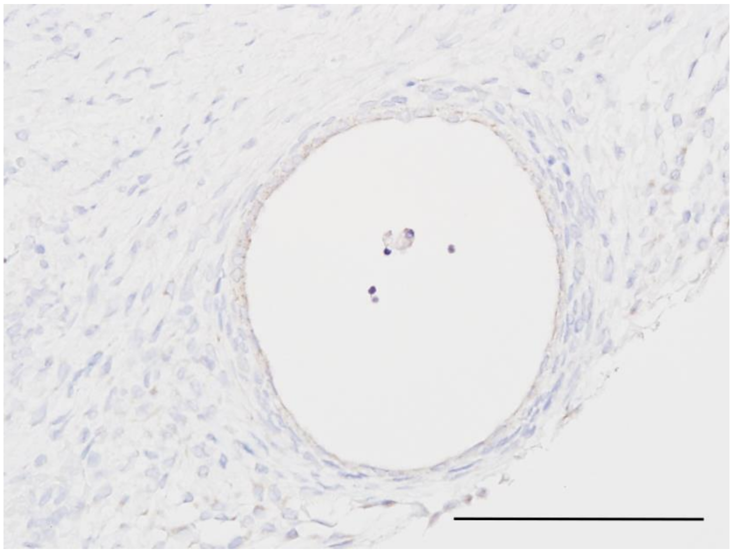

Supp. Fig. 1. No staining was observed in postmenopausal ovarian tissue sections that were exposed to normal IgG. Bars: 100 μm.

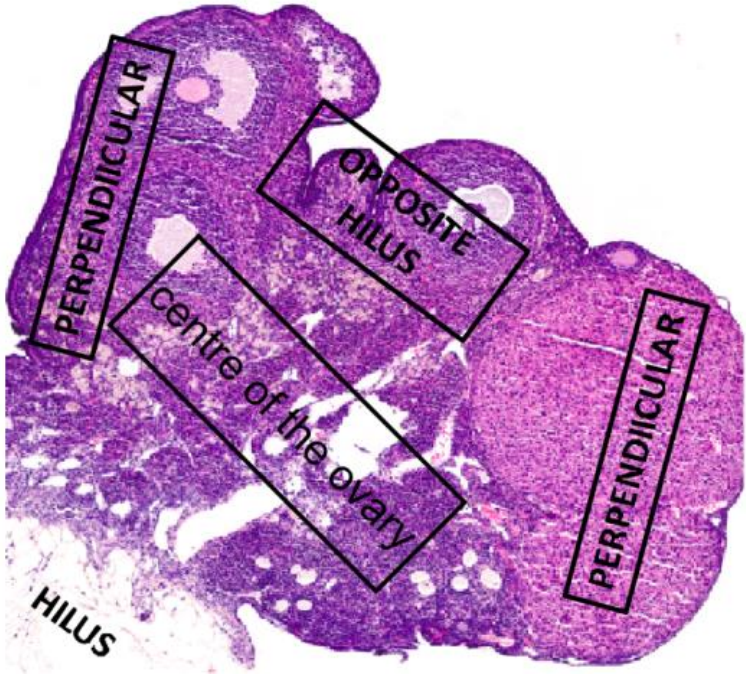

Supp. Fig. 2. Mouse ovaries were divided into four anatomical locations, namely, hilus, perpendicular to the hilus, opposite from the hilus and centre of the ovary, for the assessment of ovarian surface epithelial hyperplasia.

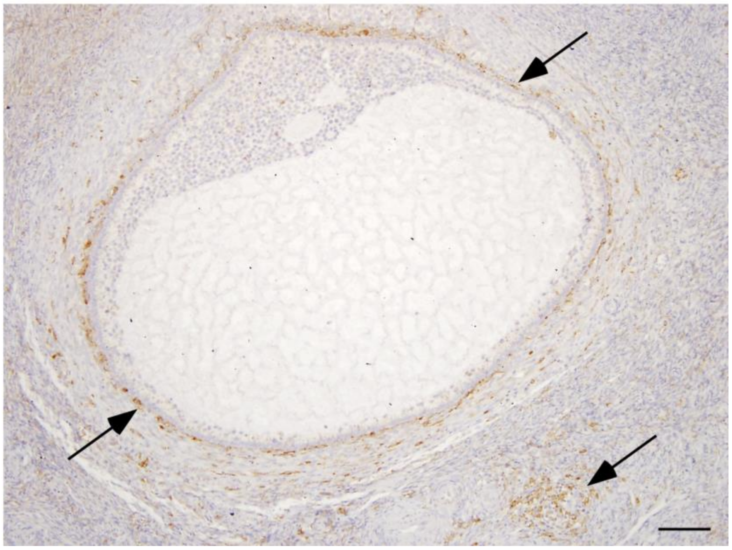

Supp. Fig. 3. A representative section from premenopausal human ovary showing pS6 immunostaining in stromal cells (arrows). Bars: 100 µm.

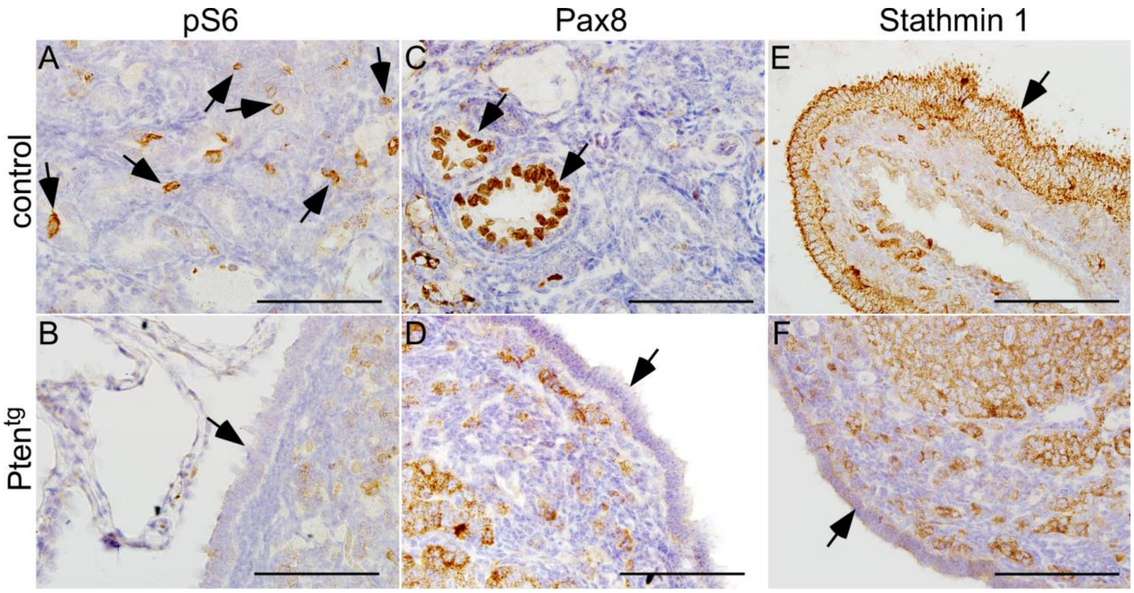

Supp. Fig. 4. Analysis of pS6, Pax8 and Stathmin 1 expression in Pten^{tg} and control mice ovaries. Arrows in panel A, C and E mark specific staining for these three markers in aged control mice ovaries. OSE (arrow in panel B, D and F) of the Pten^{tg} mice showed no expression of these markers. Bars: 100 µm.
